# Oral Microbiome Traits of Type 1 Diabetes and Phenylketonuria Patients in Latvia

**DOI:** 10.3390/microorganisms11061471

**Published:** 2023-05-31

**Authors:** Iveta Abola, Dita Gudra, Maija Ustinova, Davids Fridmanis, Darta Elizabete Emulina, Ingus Skadins, Anda Brinkmane, Una Lauga-Tunina, Linda Gailite, Madara Auzenbaha

**Affiliations:** 1 Scientific Laboratory of Molecular Genetics, Rīga Stradiņš University, LV-1007 Riga, Latvialinda.gailite@rsu.lv (L.G.); madara.auzenbaha@rsu.lv (M.A.); 2 Department of Conservative Dentistry and Oral Health, Rīga Stradiņš University, LV-1007 Riga, Latvia; 3 Latvian Biomedical Research and Study Centre, LV-1067 Riga, Latvia; dita.gudra@biomed.lu.lv (D.G.);; 4 Department of Biology and Microbiology, Rīga Stradiņš University, LV-1007 Riga, Latvia; 5 Department of Endocrinology, Children’s Clinical University Hospital, LV-1004 Riga, Latvia; una.lauga.tunina@rsu.lv; 6 Clinic of Medical Genetics and Prenatal Diagnostics, Children’s Clinical University Hospital, LV-1004 Riga, Latvia; 7 European Reference Network for Hereditary Metabolic Disorders, Children’s Clinical University Hospital, LV-1004 Riga, Latvia

**Keywords:** phenylketonuria, type 1 diabetes, microbiome, 16S rRNA sequencing

## Abstract

Some metabolic disorder treatments require patients to follow a specific diet or to consume supplements that, over time, can lead to oral microbiome alterations. Well-known disorders requiring such treatment are phenylketonuria (PKU), an inborn error of amino acid metabolism, and type 1 diabetes (T1D), a metabolic disorder that requires a specific diet regimen. Therefore, the aim of this study was to investigate the oral health and microbiome characteristics that might contribute to caries activity and periodontal disease risk in PKU and T1D patients. In this cross-sectional study, 45 PKU patients, 24 T1D patients, and 61 healthy individuals between the ages of 12 and 53 years were examined. Their anamnestic data and dental status were assessed by one dentist. Microbial communities were detected from saliva-isolated DNA using *16S rRNA* gene V3–V4 sequencing on Illumina MiSeq sequencing platform. Results revealed that the PKU patient group displayed the highest number of extracted teeth (on average 1.34), carious teeth (on average 4.95), and carious activity (44.44% of individuals) compared to the T1D and CTRL groups. The lowest numbers of filled teeth (on average 5.33) and extracted teeth (on average 0.63) per individual were observed in T1D patients. Gingivitis appeared more often in the T1D group; however, possible risk of periodontal disease was seen in both the T1D and PKU patient groups. The highest number of differentially abundant genera was detected in the PKU group (*n* = 20), with enrichment of *Actinomyces* (*p*_adj_ = 4.17 × 10^−22^), *Capnocytophaga* (*p*_adj_ = 8.53 × 10^−8^), and *Porphyromonas* (*p*_adj_ = 1.18 × 10^−5^) compared to the CTRL group. In conclusion, the dental and periodontal health of PKU patients was found to be significantly inferior compared to T1D patients and healthy controls. T1D patients showed early signs of periodontal disease. Several genera that correlate with periodontal disease development were found in both groups, thus suggesting that T1D and PKU patients should seek early and regular dental advice and be educated about proper oral hygiene practices.

## 1. Introduction

The human oral cavity consists of several intricate anatomical structures that harbor thousands of different microorganism species. Although these microorganisms are predominantly bacteria, they also include viruses, fungi, archaea and, in smaller quantities, protozoa. For this complex ecosystem to maintain a healthy balance, its environment must be consistently humid, with a temperature between 35 and 37 °C and a neutral pH [1]. The oral microbiome plays a significant role in maintaining health, both locally and systemically. With the advent of next-generation sequencing (NGS) technologies, the oral microbiome can now be extensively studied, providing important information on the varieties and quantities of bacteria found in a sample and their roles in a particular ecosystem [2,3]. Saliva is often collected for microbiome analysis as it contains numerous microorganisms that shed from various intraoral surfaces such as the teeth, gingival crevices, dorsum of the tongue, and buccal mucosa [4,5], which is an easy non-invasive sampling method and does not cause discomfort to the patient. Specific alterations in the composition of the salivary microbiome can be an indicator of different systemic conditions in the host [6,7]. Furthermore, different external factors such as oral hygiene and dietary habits can influence the oral microbiome [8,9].

Some treatments for metabolic disorders require patients to follow a specific diet or consume supplements. However, over long periods of time, these types of treatments can lead to oral microbiome alterations. One such disorder is phenylketonuria (PKU), an inborn error of amino acid metabolism caused by the defective hydroxylation of phenylalanine (Phe) to tyrosine due to very low or absent activity of the enzyme Phe hydroxylase (PAH) [10]. This PAH deficiency leads to Phe build-up in blood and tissue, most commonly resulting in severe intellectual disability if not treated [11]. The treatment of PKU entails following a strict low-protein diet and consumption of a Phe-free amino acid formula, which is usually consumed approximately three to four times a day depending on individual requirements [12]. These supplements are often sweetened with carbohydrate-containing sweeteners to make them more palatable [13]. During periods of illness, it is recommended that PKU patients consume supplementary high-energy snacks, for example, those containing glucose polymers. Frequent intake of high-glucose and/or -sucrose snacks throughout the day results in recurrent drops in salivary pH, which, combined with insufficient oral hygiene, can result in an increased incidence of caries [14]. Therefore, the specific nutritional needs of PKU patients and the use of amino acid supplements are significant factors that have the potential to influence their teeth and oral health and, consequently, their overall microbiome composition.

Another metabolic disorder that requires patients to follow a specific diet is type 1 diabetes (T1D). In contrast to patients with PKU, T1D patients must restrict their carbohydrate intake to maintain normal blood sugar levels [15]. T1D is caused by defective insulin secretion and/or action. Advancements in insulin therapy have made it increasingly easy to control the disease, to the point where a diabetic patient can now have a diet somewhat similar to that of a healthy individual [16]. However, diabetes mellitus is one of the diseases characterized by distinct alterations in the oral microbiome [17,18]. It is common for T1D patients to have altered salivary gland functions, which can influence the composition of saliva and salivary flow rate and ultimately the oral microbiome [19,20]. The available literature on oral microbiome alterations in T1D patients is limited. Although recent studies have unequivocally confirmed that the oral microbiome in T1D patients differs from that of control subjects, there is conflicting information regarding the exact nature of these differences and their clinical significance. Patients with T1D have an increased risk of periodontitis; however, at present, the oral microbiome changes in T1D patients cannot be specifically defined from the literature due to the contradictory conclusions of different studies [21,22]. These discrepancies may be the result of samples being collected from different locations in the oral cavity in different studies and/or different stages of the disease in the studied patients. It is essential to continue investigating this topic and to expand the criteria for patient participation in studies.

In this regard, the objective of the present study was to investigate the oral microbiome characteristics and oral health parameters that influence caries activity and periodontal disease risk in PKU and T1D patients compared to healthy individuals in Latvia.

## 2. Materials and Methods

### 2.1. Ethics Approval

Approval for this study was granted by the Central Medical Ethics Committee (No. 1/19-03-26) and the Genome Research Council of Latvia prior to data collection. The study was conducted according to the Helsinki Declaration. All possible risks, objectives, and benefits of involvement in the study were carefully explained to every participant, or to the parents or legal guardians of underage participants and mentally disabled patients. For participants under the age of 18 to be enrolled in the study, the parent or legal guardian provided informed consent on behalf of the child.

### 2.2. Study Design and Saliva Sample Collection

In total, 45 PKU patients (57.8% females and 42.2% males), 24 T1D patients (45.8% females and 54.2% males), and 61 healthy individuals (65.6% females and 34.4% males) between the ages of 12 and 53 years were enrolled in this cross-sectional study through the Riga Stradins University, Stomatology Institute clinic. All patients met the relevant criteria for participation in the study. Specifically, the selection criteria for T1D patients were as follows: T1D diagnosed before the age of 5, compensated diabetes (HbA1c ≤ 7%), no proliferative diabetic neuropathy, no diabetic nephropathy (GFR < 60 mL/min), following the diet recommendations (18–22 bread units—where one bread unit is equal to 12 g of carbohydrates) and no diabetic autonomic neuropathy. Exclusion criteria were not following the diet and having unsatisfactory disease control. The selection criterion for PKU patients was a definite diagnosis of PKU (PKU screening test using a few drops of blood from a newborn’s heel. The blood sample is tested in a laboratory to determine if it has too much phenylalanine in it.) [23]. The inclusion criteria for patients in the control group were as follows: healthy individuals matched by age and gender with the T1D and PKU groups, not following any specific diet, no chronic diseases, and seen for regular dentist checkups.

All study participants were instructed to have breakfast and brush their teeth in the morning as usual but not later than 2 h before saliva sample collection. Upon arrival at the Riga Stradins University Stomatology Institute Clinic, the participants (or their representatives) completed a questionnaire (Table S1), followed by sample collection. The participants were provided with a graduated plastic tube for sample collection and a quiet, private space. Saliva samples (10 mL) were taken using the unstimulated draining method; the participants were instructed to keep spitting into the tube until the 10 mL mark was reached. The samples were then centrifuged within an hour after collection at 1000 rpm for 15 min to separate epithelial cells and bacteria from the cell-free solution; only aqueous solution was removed and 3–5 mL solution was stored at −80 °C prior to DNA extraction [24].

### 2.3. Clinical Examination of Teeth and Periodontal Tissues

Salivary sample collection was followed by clinical examination to evaluate oral health. Examination was carried out by the same dentist for all patients under appropriate and uniform lighting conditions. Dental status was assessed by identifying decayed teeth, missing teeth, and filled surfaces of teeth using visuo-tactile dental examination with a sharp dental probe, mirror, 3–1 syringe, and dental magnifying loupes worn by the dentist. Several indexes were used to assess oral hygiene and gingival health: CPITN index, plaque index, and the Greene–Vermillion index, which was calculated upon detection of the presence and abundance of plaque, calculus for specific teeth, the depth of gingival sulci and/or periodontal pockets, and evaluation of gingival bleeding on probing with a periodontal probe. For the CPITN index, the presence of gum bleeding (score 1), the presence of tooth calculus (score 2), the presence of 4–5 mm periodontal pockets (score 3) and the presence of periodontal pockets of 6 mm and above (score 4) were determined. Patients with CPITN 1 and 2 scores were labeled as having gingivitis, while patients with CPITN 3 and 4 scores were classified as being at possible risk of periodontitis disease. Ten permanent teeth were examined (d17, 16, 11, 26, 27, 36, 37, 31, 46, 47) [25].

The scores from the four areas of the tooth were added together and divided by four to determine the plaque index for the tooth with the following criteria: grade 0—no plaque; grade 1—thin plaque layer at the gingival margin, only detectable by scraping with a probe; grade 2—moderate layer of plaque along the gingival margin, interdental spaces plaque free; grade 3—abundant plaque along the gingival margin, interdental spaces filled with plaque [26].

The Greene-Vermillion index is a simplified oral hygiene index with plaque and calculus components. Six tooth surfaces were scored, four posterior and two anterior (d16, 26, 11, 46, 36, 31). Plaque was scored on a scale of 0 to 3. Calculus deposits were scored for the same surfaces on a scale of 0 to 3. The index values were calculated from the recordings of the calculus and plaque scores [27]. Debris component scores: 0—no debris or stain present; 1—soft debris covering not more than one-third of the exposed tooth surface; 2—soft debris covering more than one-third but not more than two-thirds of the exposed tooth surface; 3—soft debris covering more than two-thirds of the exposed tooth surface. Calculus component scores: 0—no calculus present; 1—supra-gingival calculus covering not more than one-third of the exposed tooth surface; 2—supra-gingival calculus covering more than one-third but not more than two-thirds of the exposed tooth surface; 3—supra-gingival calculus covering more than two-thirds of the exposed tooth surface.

To compare all three groups, descriptive statistics were used with Fisher’s exact test and unpaired *t* test.

### 2.4. DNA Extraction

Thawed saliva samples were transferred to Lysing Matrix E tubes (MP Biomedicals, Irvine, CA, USA) and homogenized using a MP Biomedicals’ FastPrep-24™, Irvine, CA, USA, bead-beating system at a speed setting of 6.0 for 40 s. The samples were then centrifuged at 14,000× *g* for 10 min and the supernatant was used for DNA extraction using the phenol–chloroform method described by Rovite et al. [28]. DNA quantity was assessed using a Qubit dsDNA HS Assay Kit and a Qubit 2.0 Fluorometer (Thermo Fisher Scientific, Waltam, MA, USA).

### 2.5. 16S rRNA Gene V3–V4 Amplification and Illumina MiSeq Sequencing

A two-stage PCR protocol was applied for MiSeq library preparation. Primers were designed for PCR amplification of the *16S rRNA* gene V3–V4 region specific to the domain Bacteria [29] with Illumina overhang adapters. Each PCR reaction batch contained negative controls to monitor the purity of the reaction. Microbial DNA (4 ng) was amplified separately with V3 and V4 primers using Phusion U Multiplex PCR Master Mix (Thermo Fisher Scientific) under the following reaction conditions: denaturation at 98 °C for 30 s, 35 cycles of 98 °C for 10 s, 67 °C for 15 s, 72 °C for 15 s, and fragment elongation at 72 °C for 7 min. The yield of the acquired PCR products was assessed using 1.2% agarose gel electrophoresis and the products were purified using a NucleoMag NGS Clean-Up and Size Select kit (Macherey-Nagel, Duren, Germany). The concentration of the PCR products was measured using a Qubit dsDNA HS Assay Kit (Thermo Fisher Scientific, Waltham, MA, USA) and a Qubit 2.0 Fluorometer (Thermo Fisher Scientific, Waltham, MA, USA) and the samples were normalized to 4 ng/µL. During the second PCR stage, Illumina MiSeq i7 and i5, (Illumina Inc., San Diego, CA, USA) indexes were added to the 4 ng of V3 and V4 PCR product using custom-ordered Nextera XT Index Kit (Illumina Inc., San Diego, CA, USA) primers (Metabion International AG, Planegg/Steinkirchen, Germany). For this reaction, Phusion U Multiplex PCR Master Mix was used under the same thermal cycler reaction conditions specified for the first PCR stage. The *16S rRNA* PCR products were then pooled and purified for the sequencing reaction using NucleoMag magnetic beads. The quality and acquired amount of the *16S rRNA* V3–V4 amplicons were assessed using an Agilent High Sensitivity DNA Chip kit and Agilent 2100 BioAnalyzer (Agilent Technologies, Santa Clara, CA, USA) and a Qubit dsDNA HS Assay Kit and Qubit 2.0 Fluorometer, respectively (Table 1).

Prior to sequencing, all samples were pooled at equal molarities and diluted to 6 pM. They were then paired-end sequenced using a 500-cycle MiSeq Reagent Kit v2 and an Illumina MiSeq (Illumina Inc.). Each run was expected to produce at least 10,000 reads per sample. After the sequencing run was completed, the individual sequence reads were filtered using MiSeq Software v.4.0 (Illumina, San Diego, CA, USA) to remove low-quality sequences. All MiSeq quality-approved, trimmed, and filtered data were exported as fastq files.

### 2.6. 16S Sequence Analysis

Sequence reads were demultiplexed using Illumina’s MiSeq Reporter Software v.2.6 (Illumina, San Diego, CA, USA) and quality-filtered using Trimmomatic [30] v.0.39 with the leading quality of Q20 and trailing quality of Q20, and sequences shorter than 36 nucleotides were discarded. All quality-approved sequences were imported into the QIIME 2 [31] v.2021.11 environment for further analysis. The DADA2 [32] plug-in was used to pair forward and reverse reads, as well as for extra sequence quality control and chimeric sequence removal using a pooled consensus method. The resulting feature table and sequences were used for de novo clustering employing the VSEARCH plug-in and using a 97% identity threshold [33]. Thereafter, de novo multiple-sequence alignment was performed using the MAFFT method [34], while phylogenetic trees were constructed using FastTree 2 [35]. De novo clustered sequences were used for taxonomic assignment based on the expanded Human Oral Microbiome Database v.3 (RefSeq v.15.22) identity reference database that was compiled using the RESCRIPt tool [36].

### 2.7. Statistical Analysis

Initially, samples were rarefied to an even sequencing depth of 10,820 sequences per sample and rarefaction curves were built using the vegan v.e.6-2 package. Thereafter, species richness measurements (observed operational taxonomic units (OTUs), (Chao1), alpha diversity (Shannon, Simpson), and beta diversity (weighted and unweighted UniFrac metrics, Jaccard distance) were calculated using the phyloseq package [37] within the R environment. To assess the significance of alpha diversity measurements between study groups, the Wilcoxon rank sum test and the Holm *p*-value adjustment method were performed using the vegan package. Additionally, ANOVA-like pairwise comparison permutation tests were conducted to assess the significance of each study group within each of the UniFrac metrics.

Next, to evaluate the overlaps of bacterial genera between study groups, a Venn diagram was constructed using ggVennDiagram [38] v.0.1.9 within the R environment.

Then, the abundances of each taxonomic entity were converted into relative abundances and the relationship between the top 40 most abundant genera and study-group-associated metadata were determined via Pearson correlation analysis with the Benjamini–Hochberg *p*-value adjustment method based on taxa and groups using the package microbiomeSeq [39] v.0.1.

To determine significant taxonomic entities across study groups with/without adherence to a diet regime, differential expression analysis based on negative binomial distribution was performed using the package DESeq2 [40] v.1.34.0 implemented in R. Taxonomic counts were normalized using log-relative transformation, and significantly differentially abundant taxa (*p* < 0.001) with |log_2_(fold change)| > 2 were visualized using the ggplot2 package.

Redundancy analysis (RDA) with the vegan package was used to extract and summarize the variation in a dataset that can be explained by the explanatory variables. Each variable was tested using ANOVA analysis, and a further reduction in the number of explanatory variables entering the analysis was performed via Monte Carlo permutation test based on the Akaike information criterion and *p*-values for the comparison of the variable.

## 3. Results

### 3.1. Group Information

A total of 130 samples were available for the microbiome analyses based on sequencing quality criteria from the three study groups: type 1 diabetes patients (T1D group, 24 samples), phenylketonuria patients (PKU group, 45 samples), and generally healthy people as the control group (CTRL group, 61 samples). Table 2 presents a characterization of all three groups, including oral hygiene habits, calculus removal necessity, and periodontal status. Within the PKU group, strict adherence to the diet regiment was reported for 27 individuals, partial adherence to the diet regimen was reported for 11 individuals, and 7 individuals did not follow the diet regimen. In the same group, most of the individuals brushed their teeth twice per day (60.0%), one-third once per day (28.89%), and a minority of individuals did not brush their teeth (11.11%). It was in the PKU group that the highest numbers of extracted teeth (on average 1.34), carious teeth (on average 4.95), and carious activity (44.44% of individuals) were observed, compared to the T1D and CTRL groups. Within the T1D group, strict adherence to diet was reported for all individuals (100%) and a majority of them brushed their teeth twice per day (62.05%), while a considerable number of individuals brushed their teeth once per day (37.5%). In comparing the three study groups, it was in the T1D group that the lowest numbers of filled teeth (on average 5.33) and extracted teeth (on average 0.63) per individual were observed. The possible risk of periodontal disease showed up in both patient groups (T1D and PKU), while gingivitis appeared more often in the T1D group.

### 3.2. Taxonomic Structure and Diversity Analysis

All samples were analyzed using *16S rRNA* V3–V4 sequencing. After quality-filtering, 7,347,913 sequences were obtained for 130 samples with an average of 56,960 ± 22,037 reads per sample. Subsequently, samples were rarefied to an even sequencing depth, resulting in 10,820 sequences and 78.5 ± 196.0 OTUs (operational taxonomic units) per sample (Figure S1).

**Figure 1:**
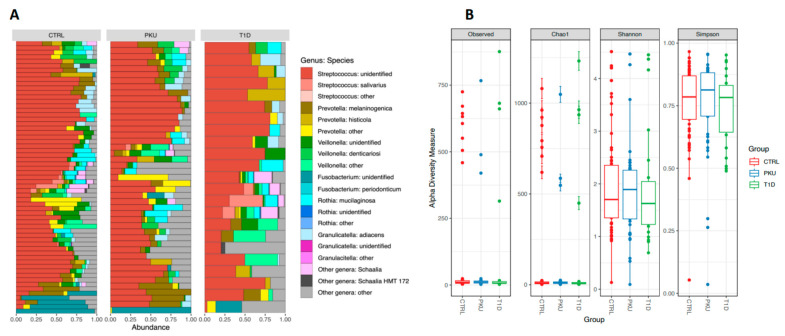
Summary of taxonomic composition and alpha diversity measurements across the three study groups. (**A**) Bar plot of taxonomic entities displaying top bacterial genera and their respective species found in CTRL, PKU, and T1D patient saliva samples. (**B**) Alpha diversity metrics were estimated for observed OTUs, Chao1, Shannon, and Simpson indexes (colored according to the study group).

The most abundant bacterial genera in the CTRL group were *Streptococcus* (55.4%), *Prevotella* (9.5%), *Veillonella* (9.1%), and *Rothia* genera (4.5%). Similarly, the dominant genera in the T1D and PKU groups were *Streptococcus* (T1D: 53.3%, PKU: 48.5%), *Veillonella* (T1D: 10.3%, PKU: 8.2%), *Prevotella* (T1D: 9.1%, PKU: 12.6%), and *Rothia* (T1D: 4.7%, PKU: 5.5%) (Figure 1A). Considering the abundance at the species level, the most common bacterial species in the CTRL group were unidentified *Streptococcus* species (54.1%), *Prevotella melaninogenica* (5.3%), unidentified *Veillonella* species (4.5%), and *Rothia mucilaginosa* (4.4%). The most common bacterial species in the T1D group were unidentified *Streptococcus* species (16.3%), unidentified *Pseudomonas* species (4.9%), *Veillonella atypica* (4.5%), and *Rothia mucilaginosa* (4.5%), whereas in the PKU group they were unidentified *Streptococcus* species (48.3%), *Prevotella melaninogenica* (8.0%), *Rothia mucilaginosa* (5.5%), and unidentified *Pseudomonas* species (4.6%).

Pairwise comparisons showed that alpha diversity measurements—observed OTUs (number of taxonomic groups observed in the sample), Chao1 (total richness), Shannon index (mean species diversity), and Simpson index (mean species evenness)—did not differ significantly among the three study groups (*p* > 0.05, Figure 1B, Table S2), suggesting similar intrasample diversity levels. The PKU group was analyzed separately in relation to adherence to diet (strict, partial, or no diet regime). It was found that none of the estimated diversity indexes differed significantly in this regard (*p* > 0.05, Table S2). The CTRL and T1D groups were not subjected to this analysis as the CTRL subjects had no diet restrictions and all the T1D patients followed a strict diet.

**Figure 2:**
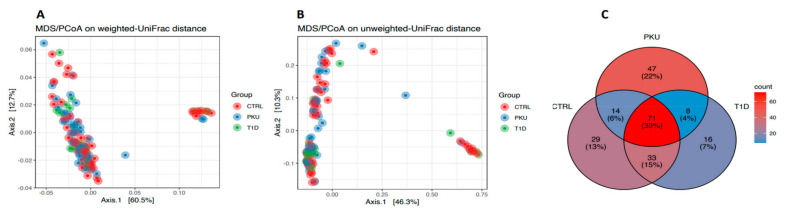
Beta diversity and quantity of bacterial species shared among the PKU, T1D, and CTRL groups. Principal coordinate analysis (PCoA) of the salivary microbiome samples estimated via (**A**) weighted UniFrac, (**B**) unweighted UniFrac, and (**C**) Venn diagram of shared species across the three study groups.

To examine whether differences existed in intersample variability among the PKU, T1D, and CTRL groups, pairwise comparisons of UniFrac metrics distance were carried out. Weighted and unweighted UniFrac metrics (Figure 2A,B) of the salivary microbiome at the genus level by study group showed no significant differences (*p*_adj_ > 0.05, with 999 permutations, Table S3) in beta diversity among the three groups, suggesting that the global compositions of bacterial communities across our study groups were highly similar. These results were supported by a Venn diagram (Figure 2C) showing that the intersection (common to all three study groups) had the highest number of species (*n* = 71, corresponding to 33%). The lowest number of shared species between two groups was for the PKU and T1D groups (*n* = 8, corresponding to 4%), including such entities as *Prevotella* HMT 317, *Eubacterium nodatum*, *Prevotella intermedia*, *Alloprevotella tannerae*, and others. The highest number of shared species between two groups was for the T1D and CTRL groups (*n* = 33, corresponding to 15%), including such entities as *Eubacterium saphenum*, *Solobacterium moorei*, *Filifactor alocis*, *Oribacterium asaccharolyticum*, *Capnocytophaga gingivalis*, and others. The highest number of unique species belonged to the PKU group (*n* = 47, corresponding to 22%), including such exemplars as *Dialister pneumosintes*, *Selenomonas noxia*, *Bifidobacterium breve*, *Schaalia odontolyticus*, *Mycoplasma salivarium*, and others.

### 3.3. Salivary Microbiome Differences among the Groups

Differential analysis was used to investigate the bacterial entities that differed significantly between the PKU and T1D groups. Differential abundance testing with DESeq2 (*p*_adj_ < 0.001, |log_2_(fold change)| > 2) revealed four species that were differentially abundant across the PKU, T1D, and CTRL groups, namely an unidentified *Leptotrichia* species (*p*_adj_ = 1.15 × 10^−15^), *Prevotella pallens* (*p*_adj_ = 4.88 × 10^−12^), an unidentified *Fusobacterium* species (*p*_adj_ = 8.05 × 10^−5^), and an unidentified *Capnocytophaga* species (*p*_adj_ = 1.19 × 10^−4^) (Figure S2).

Differential analysis was also used to investigate which bacterial species significantly differed between the saliva samples of the T1D and CTRL groups (Figure S3). Six species were found to differ significantly between the samples of these two groups, with species enriched in the T1D group including unidentified *Leptotrichia* species (*p*_adj_ = 9.65 × 10^−9^) and unidentified *Porphyromonas* species (*p*_adj_ = 2.57 × 10^−5^), whereas depleted species within the T1D group included *Prevotella pallens* (*p*_adj_ = 2.14 × 10^−10^), an unidentified *Fusobacterium* species (*p*_adj_ = 8.04 × 10^−5^), *Veillonella denticariosi* (*p*_adj_ = 8.04 × 10^−5^), and *Gemella haemolysans* (*p*_adj_ = 1.21 × 10^−4^). Furthermore, saliva samples from the PKU and CTRL groups underwent a similar analysis. The abundance of 17 bacterial species-level entities was found to differ significantly between the PKU and CTRL groups. Enriched species in the PKU group included such entities as *Veillonella dispar* (*p*_adj_ = 9.44 × 10^−16^), *Haemophilus pittmaniae* (*p*_adj_ = 2.16 × 10^−4^), and others, while depleted species included *Streptococcus salivarius* (*p*_adj_ = 5.81 × 10^−31^), *Prevotella pallens* (*p*_adj_ = 9.53 × 10^−4^ *Prevotella salivae* (*p*_adj_ = 2.31 × 10^−14^), *Rothia dentocariosa* (*p*_adj_ = 4.08 × 10^−9^), *Megasphaera micronuciformis* (*p*_adj_ = 1.08 × 10^−8^), and others (Figure S4). Furthermore, saliva samples from the PKU group were separated according to the diet regime (strict, partial, or no diet regime) and subjected to differential analysis together with CTRL group samples. As a result, 24 bacterial species were found to differ significantly among the three PKU groups and the CTRL group. These included species such as *Streptococcus salivarius* (*p*_adj_ = 1.91 × 10^−25^), which was depleted in all PKU groups but its abundance was slightly higher in individuals with a strict diet regime; *Schaalia* HMT-172 (*p*_adj_ = 2.15 × 10^−14^), which was depleted in all PKU groups, but among the diet groups, its abundance was higher in individuals with partial adherence to the diet; *Veillonella dispar* (*p*_adj_ = 2.42 × 10^−11^), which was enriched in all PKU groups but the highest abundance was observed in individuals with partial adherence to the dietary regimen; *Rothia dentocariosa* (*p*_adj_ = 5.08 × 10^−10),^ which was depleted in all PKU groups but had the highest abundance in the strict diet group; *Prevotella salivae* (*p*_adj_ = 7.44 × 10^−10^), which was depleted in all PKU groups but had the highest abundance in the partial diet adherence group; *Porphyromonas gingivalis* (*p*_adj_ = 1.64 × 10^−7^), which was enriched only in individuals following a strict diet regimen; *Porphyromonas pasteri* (*p*_adj_ = 2.57 × 10^−6^), which was depleted in all PKU groups; and others (Figure S5).

### 3.4. Correlations between Salivary Bacteria and Clinical Parameters

**Figure 3:**
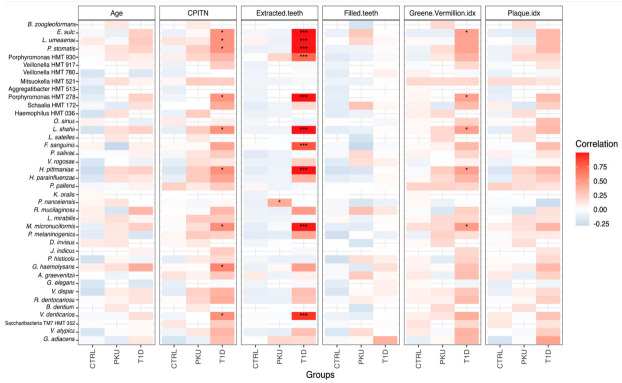
Correlation map of taxonomic entities at the species level with patient-specific characteristics across the CTRL, PKU, and T1D study groups. * *p* = (0.001, 0.05]. *** *p* = [0, 0.001].

To evaluate the associations of patient-specific characteristics such as age, carious teeth, extracted teeth, plaque index, and others with the taxonomic entities of salivary microbiomes across the study groups, correlation analysis was carried out (Figure 3). Upon testing each variable within the PKU group, we found only one significant correlation—a positive correlation of *Prevotella nanceiensis* with the number of extracted teeth (*p*_adj_ = 0.038, *r* = 0.43). On the other hand, many species showed significant positive correlations with patient-specific characteristics in the T1D group; for instance, nine species showed a significant positive correlation with CPITN, including *Peptostreptococcus stomatis* (*p*_adj_ = 0.029, *r* = 0.54), *Haemophilus pittmaniae* (*p*_adj_ = 0.024, *r* = 0.55), *Megasphaera micronuciformis* (*p*_adj_ = 0.017, *r* = 0.57), *Gemella haemolysans* (*p*_adj_ = 0.018, *r* = 0.6), and *Veillonella denticariosi* (*p*_adj_ = 0.019, *r* = 0.56); additionally, ten species were found to have a significant positive correlation with the number of extracted teeth in the T1D group, which included such species as *Lachnoanaerobaculum umeaense* (*p*_adj_ = 5.32 × 10^−17^, *r* = 0.99), *Peptostreptococcus stomatis* (*p*_adj_ = 1.95 × 10^−21^, *r* = 0.99), *Fastidiosipila sanguinis* (*p*_adj_ = 2.58 × 10^−5^, *r* = 0.8), *Haemophilus pittmaniae* (*p*_adj_ = 3.54 × 10^−23^, *r* = 0.99), *Porphyromonas* HMT_930 (*p*_adj_ = 0.00075, *r* = 0.72), *Megasphaera micronuciformis* (*p*_adj_ = 2.56 × 10^−18^, *r* = 0.99), *Veillonella denticariosi* (*p*_adj_ = 3.07 × 10^−9^, *r* = 0.92), and others. In addition, within the T1D group, the species *Megasphaera micronuciformis* (*p*_adj_ = 0.041, *r* = 0.49) was found to have a significant positive correlation with the Greene–Vermillion index.

In contrast to the results for the PKU and T1D study groups, we were unable to identify any taxonomic entity that was significantly associated with any of the patient-specific characteristics for the CTRL group.

Redundancy analysis (RDA) was conducted to extract and summarize the variation in the dataset that could be explained by the explanatory variables. To do so, the dataset was divided into two parts: a CTRL and T1D dataset, and a CTRL and PKU dataset. In the joint dataset of T1D and CTRL, upon testing each variable, ANOVA analysis identified “Group” (*p* = 0.002, 999 perm.) as a significant determinant of the microbial composition. We then reduced the number of explanatory variables entering the analysis to optimize the variations explained by them. However, all other patient-characterizing variables, such as CPITN (*p* = 0.175, 999 perm.), number of extracted teeth (*p* = 0.24, 999 perm.), Greene–Vermillion index (*p* = 0.77, 999 perm.), carious teeth (*p* = 0.77, 999 perm.), plaque index (*p* = 0.87, 999 perm.), age (*p* = 0.51, 999 perm.), and others, were insignificant determinants of the microbial community in relation to the study group (Figure S6). The results were confirmed by testing the parsimonious RDA model with the global model (*p* = 0.003, 999 perm.).

In the joint PKU and CTRL dataset, upon testing each variable, ANOVA analysis again identified “Group” (*p* = 0.033, 999 perm.) as a significant determinant of the microbial composition. As in the previous joint dataset analysis, we then reduced the number of explanatory variables entering the analysis to optimize the variation explained by them. In this case, by using forward selection, the variables “Frequency of tooth brushing” and “Gender” were both identified as significant determinants (*p* = 0.005 and *p* = 0.05, respectively, 999 perm.) in shaping microbial community composition (Figure S7). The results were confirmed by testing the parsimonious RDA model with the global model (*p* = 0.009, 999 perm.).

## 4. Discussion

Prior to conducting this study, it was evident to the authors that the oral health and oral microbiome characteristics in PKU and T1D patients required more in-depth research as both groups have a long-term diet that it influences oral microbiome and oral health. On this topic, there are few studies published [22,41]. Therefore, three different groups were chosen to estimate the potential influence of specific diet on the oral microbiome. PKU patients follow a strict low-protein diet with consumption of a Phe-free amino acid formula. Additionally, their diets are often high in carbohydrates, which are usually taken throughout the day to meet their energy requirements [42]. T1D patients who were enrolled in this study followed a restricted carbohydrate intake and balanced diet. Control group individuals did not follow any special diet plan.

### 4.1. General Characterization of Oral Status in PKU, T1D, and CTRL Groups

Previous studies have shown that although children with T1D possess a lower caries risk, they have an increased risk of developing periodontal disease because of the restricted carbohydrate (sugar) intake in diabetic patients [7,10]. However, more recent research has reported no difference in the caries prevalence in children with T1D [11,12,13]. This could be explained by the fact that, over time, advancements in insulin therapy regimes have led to a better control of the disease, reducing the difference in diet regime between T1D and healthy children [14]. Patients included in our study with T1D had good glucose level control, permanent teeth, and most of them ate regular meals not enriched with carbohydrates; we found this last aspect very important for comparing the influence of dietary habits on the oral microbiome. Our study showed that T1D patients had a higher risk of gingivitis, which is a reversible stage of periodontal disease, provided that the patient follows an oral hygiene routine [43]. Ballikaya et al. [41] reported higher caries activity among PKU patients, which is in line with our study findings. In accordance with our results showing that the PKU patient group showed the highest caries activity (44.44%), da Costa Silveira et al. [44] found that 75% of PKU patients were at high risk of caries activity. Caries prevention and treatment is of major importance in the dental care of these patients. Another significant finding in our study was the greater risk of periodontal disease development in PKU patients.

The main risk factor for periodontal disease development is the constant presence of dental plaque [45]. Almost all PKU and T1D patients in the current study required plaque and calculus removal by a professional dental hygienist. Acquired evidence suggests that PKU and T1D patients are at an increased risk of dental caries and periodontal disease. In our enrolled PKU and T1D groups, patients struggle with maintaining good daily oral hygiene habits, many brushing their teeth only once a day (28.89%) and 11.11% not brushing at all, which has a strong correlation with oral health [46]. After discussions with and clinical examination of our patients, we speculated that this is also because parents initially, and later PKU patients themselves, are so focused on Phe levels and formula consumption that they do not pay enough attention to dental care, which is very important if they do not rinse their teeth after formula consumption.

Patients who visit the dentist regularly are more willing to maintain good care of their oral health, which is why it is very important to motivate PKU and T1D patients to visit their dentist and dental hygienist every 3 to 6 months. Periodontal disease should be diagnosed and treated as early as possible with professional oral hygiene procedures and periodontal treatment [47].

### 4.2. Microbiome Evaluation in PKU, T1D, and Control Groups in Association with Oral Health

With the importance of human and microbiome interactions becoming increasingly evident, greater attention is now being paid to the elucidation of the healthy states of microbiomes and to the identification of the microbiome markers that might contribute to disease development in the human host. In the case of the oral microbiome, acquisition of such knowledge could benefit the development of treatment options or probiotic guidelines for patients with unhealthy oral cavities. Therefore, this study aimed to evaluate the oral status and microbiome alterations that influence caries activity and periodontal disease risk in PKU and T1D patients compared to healthy individuals.

Our results revealed that the most dominant bacterial genus in all the collected saliva samples, regardless of the study group, was *Streptococcus,* which is frequently referenced in oral microbiome studies. Several studies suggest that *Streptococci* are a normal component of the oral microbiome, while others propose that they are associated with dental caries and other oral pathologies [48,49,50]. For example, Dianawati et al. [48] examined the distribution of *Streptococcus mutans* and *Streptococcus sobrinus* in patients with seriously high levels of dental caries and concluded that these two bacterial species are the main factors that cause dental caries. It has also been reported that *Streptococcus mutans* is the most detected bacterial species in carious lesions, consequently leading *Streptococcus mutans* to be considered the major pathogen of dental caries [48,49]. Cheon et al. [49] evaluated the association of *Streptococcus mutans* genotype diversity, commonality, and stability with dental caries history in a high-caries-risk community. They reported that lower diversity and higher stability of *Streptococcus mutans* genotypes were notably associated with fewer decayed surfaces [49]. Thus, it is well-established that oral bacteria in dental plaque biofilm are essential for the development and advancement of dental caries, one of the most prevalent chronic diseases worldwide [51]. In our study, no association with oral health was observed.

In our research on children with T1D, an increased prevalence of *Streptococcus mutans* and *Lactobacillus casei* was observed in their oral microbiome. These species are considered to be cariogenic by some researchers [21]. In our group’s work, as well as in other research, increased caries activity was not observed in the T1D group. A study by Singh-Hüsgen et al. investigated clinical oral cavity condition and the abundance of several bacterial species in children with PKU and T1D. They found that children with PKU exhibited lower levels of *Lactobacillus* species, whereas children with T1D had higher counts of *Lactobacillus* and *Streptococcus mutans*, but lower levels of *Porphyromonas gingivalis* [52].

We found that there were intersample dissimilarities between the PKU and T1D groups, as indicated by the Jaccard distance measurement. While only a limited amount of research has been conducted comparing the oral microbiomes of PKU and T1D patients with healthy subjects, some data in the literature suggest that poorer glycemic control correlates with a higher number of bacteria and an increased alpha diversity in children with T1D [52,53]. There are also differences in microbiome beta diversity depending on the site of sample collection—for example, gingival sulcus versus buccal mucosa. Jensen et al. [22] proposed that changes in alpha diversity in subgingival plaque are a marker of higher periodontitis risk in children with T1D. They found that children with more plaque and poorer oral hygiene habits had more pronounced alpha diversity. Regarding adults with T1D, it has been reported that there are marked differences in the oral microbiome, and especially in beta diversity. Several studies have concluded that T1D is characterized by a marked increase in the number of microorganisms in dental plaque [22]. In our study, we analyzed the microbiome in saliva, not in plaque, which could have affected the results, and T1D patients were not divided into children and adults.

Our results also revealed that the number of bacterial genera significantly differed among the PKU, T1D, and CTRL groups. In particular, two genera—*Alloprevotella* and *Leptotrichia*—were differentially abundant throughout all comparisons. The relative abundance of *Alloprevotella* was significantly higher in the CTRL group compared to the PKU and T1D groups, whereas the relative abundance of *Leptotrichia* was significantly higher in the T1D group. Both bacterial genera are known to be associated with oral pathologies, such as dental caries, periodontitis, and oral cancer [54,55]. In our study, association with oral health status was not observed, which could be explained by the age of our patients.

The highest number of differentially abundant bacterial genera (17 bacterial species) in the salivary microbiome was detected between the PKU and CTRL groups. When comparing patients with different diet regimes, it was observed that genera such as *Actinomyces*, *Capnocytophaga*, *Haemophilus*, and *Porphyromonas* were enriched in PKU patients who maintained a strict adherence to the diet, as compared with CTRL individuals. Furthermore, an increased abundance of *Capnocytophaga* was observed in PKU patients who maintained a partial adherence to the diet, and *Porphyromonas* enrichment was detected in PKU patients with both partial adherence to the diet and no diet regime. Of all the listed genera, only *Haemophilus* has been described as a classical oral microbiome exemplar, with an increased abundance previously being reported in healthy individuals [56,57], in contrast to our results. Moreover, recent studies have demonstrated an association of *Actinomyces* with the initiation of suppurative and granulomatous inflammatory lesions, and *Capnocytophaga* and *Porphyromonas* with periodontal disease [58,59].

A smaller number of differentially abundant bacterial genera in the salivary microbiome were observed between the T1D and CTRL groups. Differential analysis was also used to investigate which bacterial species significantly differed between the saliva samples of the T1D and CTRL groups. Six species were found to differ significantly between the samples of these two groups. *Leptotrichia* were enriched in T1D patients, while others, including *Alloprevotella* and *Fusobacterium*, were diminished. In addition to *Capnocytophaga*, the genera *Leptotrichia*, *Alloprevotella*, and *Fusobacterium* were identified as common oral microbiome constituents. Different species in this group could be related to a restricted carbohydrate diet. Furthermore, many species showed significant positive correlation with patient-specific characteristics in the T1D group; for instance, nine species showed a positive correlation with the CPITN index. As reported in the literature previously, increased abundances of these bacteria are associated with oral cancer and periodontitis [60]; therefore, follow-up with these patients is very important. If future studies confirm our results, an increased frequency of oral hygienist visits should be strongly recommended for all T1D patients.

Differential analyses have previously identified several bacterial genera that correlate with patient-specific characteristics in persons without diet restrictions, particularly *Porphyromonas* and *Fusobacterium* [61,62]. We did not identify any bacterial genus in the CTRL group that had a significant correlation with any of the patient-specific characteristics, indicating that the sample set we used was a suitable CTRL group.

We found that *Porphyromonas* was significantly enriched in PKU and T1D patients and was associated with several patient-characterizing parameters (extracted teeth and the Greene–Vermillion index). Cervino et al. reported previously that patients who suffer from both periodontitis and diabetes are prone to developing renal, cardiovascular, and ocular diabetes complications [63].

It is clear that, from a clinical perspective, it is important to diminish the persistence of these pathogens. Chlorhexidine, a common antiseptic agent with a broad-spectrum antibacterial activity, is able to reduce plaque, gingival inflammation, and bleeding [64], but because of tooth discoloration, dysgeusia, and bacterial recolonization, it is not recommended for use over a long period [65]. Based on this information, we would strongly recommend herbal mouthwashes as home prophylactic measures [66]. For instance, mouthwashes containing curcumin and the peppermint plant have antibacterial and bacterial growth inhibition properties when used to treat periodontal disease [67].

One limitation of this study was the small number of included patients and their uneven age (the T1D group was younger than the PKU and CTRL groups), as striking differences in the number of genera significantly associated with the patient-specific characteristics between the PKU and T1D groups could at least partly be attributed to the uneven number of subjects in each study group, thus affecting the statistical power. The results of this study illustrate the need for further studies with larger sample sizes to understand the full picture of how PKU dietary restrictions and treatments affect patients over time. Another limitation is related to the low number of OTUs present in the current sample set. Since saliva is known for its high enzymatic activity, these samples should be processed according to a well-standardized workflow that involves specialized sample preservation procedure. Therefore, it is plausible that a proportion of DNA was lost during sample storage, resulting in a decrease in the number of OTUs. Although the obtained results coincide well with the dental hygiene results of the participants of this study and the results of other studies related to the oral microbiome, increased attention should be paid to the standardization of storage conditions, sample processing and analysis, which could collectively result in a higher number of OTUs.

In summary, this study provides an important overview of the clinical situation of PKU and T1D patients, especially since there are very few sources in the literature discussing the oral health of PKU patients and there are no specific recommendations for their oral hygiene. The weakness of this study is related to checking whether PKU patients follow the strict diet as they claim. PKU patients taking the blood tests once a month cannot be truly indicative of the situation. As Latvia is a very small country, and it is difficult to make valuable conclusions from the small patient count, we hope that our study will encourage other colleagues to perform similar studies to help provide the best possible care for these patients.

## 5. Conclusions

Overall, PKU patients had significantly worse dental condition than healthy controls and T1D patients, and they were observed to have the highest microbial diversity associated with oral hygiene status.

PKU patients had the highest amounts of *Actinomyces, Capnocytophaga*, and *Porphyromonas*, whereas T1D patients had the highest quantities of the pathogens *Leptotrichia* and *Prevotella*, which directly correlate with periodontal disease development. To prevent periodontal disease, PKU and T1D patients should see a professional dental hygienist every 3 to 6 months.

This study can act as a guide for further studies to help PKU and T1D patients with recommendations to prevent periodontal diseases and to reduce caries risk.

## Figures and Tables

**Table 1:** The primers used in this study with Illumina overhang adapters to amplify the 16S V3–V4 region.

Name	Sequence (Illumina Adapter, Heterogeneity Spacer, 16S Region Primer)	Reference
16S V3 Fw (341F)	TCGTCGGCAGCGTCAGATGTGTATAAGAGACAGNNNNNNCCTACGGGNGGCWGCAG	[29]
16S V4 Rs (805R)	GTCTCGTGGGCTCGGAGATGTGTATAAGAGACAGNNNNNNGACTACHVGGGTATCTAATCC	[29]

**Table 2:** PKU, T1D, and CTRL patient group characteristics, hygiene habits, and oral status.

		PKU (*n* = 45)	T1D (*n* = 24)	CTRL (*n* = 61)	*p*-Value of PKU vs. CTRL	*p*-Value of T1D vs. CTRL	*p*-Value of PKU vs. T1D
Gender (male *n* (%))		19 (42.22%)	13 (54.17%)	21 (34.43%)	0.4259	0.1394	0.4483
Age (average in years ± SD)		24.78 ± 9.98	18.42 ± 9.98	27.48 ± 10.2	0.1770	0.0004	0.0141
Adherence to diet	Strict, *n* (%)	27 (60.0%)	24 (100%)	0	NA	NA	NA
	Partly, *n* (%)	11 (24.44%)	0	0	NA	NA	NA
	No diet regime, n (%)	7 (15.56%)	0	61 (100%)	NA	NA	NA
Frequency of tooth brushing	Twice per day, *n* (%)	27 (60.0%)	15 (62.5%)	58 (95.08%)	<0.0001	0.0004	1
	Once per day, *n* (%)	13 (28.89%)	9 (37.5%)	2 (3.28%)	0.0003	0.0001	0.5888
	Do not brush, *n* (%)	5 (11.11%)	0	0	0.0121	1	0.1550
	No information, *n* (%)	0	0	1 (1.64%)	NA	NA	NA
Filled teeth (average ± SD)		5.83 ± 5.06	5.33 ± 5.05	8.27 ± 5.05	0.0157	0.0179	0.6969
Extracted teeth (average ± SD)		1.34 ± 2.35	0.63 ± 2.36	0.73 ± 2.39	0.1937	0.8621	0.2369
Carious teeth (average ± SD)		4.95 ± 3.7	2.79 ± 3.52	1.92 ± 3.42	0.0001	0.2981	0.0218
Caries activity	High, *n* (%)	20 (44.44%)	5 (20.83%)	6 (9.84%)	<0.0001	0.2790	0.0677
	Medium, *n* (%)	15 (33.33%)	12 (50.0%)	20 (32.79%)	1	0.2132	0.2033
	Low, *n* (%)	6 (13.33%)	7 (29.17%)	34 (55.74%)	<0.0001	0.0323	0.1942
	No information, *n* (%)	4 (8.89%)	0	1 (1.64%)	NA	NA	NA
Calculus removal	Not Required, *n* (%)	3 (6.67%)	7 (29.17%)	31 (50.82%)	<0.0001	0.0915	0.0261
	Required, *n* (%)	38 (84.44%)	17 (70.83%)	29 (47.54%)	<0.0001	0.0585	0.2166
	No information, *n* (%)	4 (8.89%)	0	1 (1.64%)	NA	NA	NA
CPITN index	0, *n* (%)	1 (2.22%)	3 (12.5%)	28 (45.9%)	<0.0001	0.0053	0.1176
	1, *n* (%)	11 (24.44%)	16 (66.67%)	18 (29.51%)	0.6613	0.0028	0.0009
	2, *n* (%)	13 (28.89%)	3 (12.5%)	13 (21.31%)	0.438	0.5389	0.1472
	3, *n* (%)	10 (22.22%)	2 (8.33%)	1 (1.64%)	0.0007	0.1909	0.1935
	4, *n* (%)	6 (13.33%)	0	0	0.0048	1	0.0850
	No information, *n* (%)	4 (8.89%)	0	1 (1.64%)	NA	NA	NA
Plaque index	0, *n* (%)	1 (2.22%)	5 (20.83%)	26 (42.62%)	0.0001	0.0806	0.0171
	1, *n* (%)	11 (24.44%)	9 (37.5%)	19 (31.15%)	0.5167	0.6140	0.2775
	2, *n* (%)	13 (28.89%)	5 (20.83%)	13 (21.31%)	0.4938	1	0.5715
	3, n (%)	16 (35.56%)	5 (20.83%)	2 (3.28%)	<0.0001	0.0175	0.2755
	No information, *n* (%)	4 (8.89%)	0	1 (1.64%)	NA	NA	NA
Greene–Vermillion index	0, *n* (%)	3 (6.67%)	4 (16.67%)	27 (44.26%)	<0.0001	0.0237	0.2268
	1, *n* (%)	9 (20.0%)	10 (41.67%)	20 (32.79%)	0.1873	0.4597	0.0882
	2, *n* (%)	16 (35.56%)	9 (37.5%)	12 (19.67%)	0.0779	0.1003	1
	3, *n* (%)	13 (28.89%)	1 (4.17%)	1 (1.64%)	<0.0001	0.4874	0.0249
	No information, *n* (%)	4 (8.89%)	0	1 (1.64%)	NA	NA	NA

Abbreviations: PKU—patients of phenylketonuria group; T1D—patients of diabetes mellitus type 1 group; CTRL—control group of generally healthy individuals; SD—standard deviation, and NA—unrepresentable values.

## Data Availability

Raw sequencing data have been deposited in the European Nucleotide Archive under study accession No. PRJEB48982.

## Supplementary Materials

The following supporting information can be downloaded at: https://www.mdpi.com/article/10.3390/microorganisms11061471/s1, Figure S1: Rarefaction curves before sample rarefaction (A) and after rarefying samples to an even sequencing depth—10,820 sequences per sample (B); Figure S2: Differentially abundant species-level entities across the study groups at the significance cut-off of *p* < 0.001; Figure S3: Differentially abundant species-level entities across the CTRL and T1D study groups at the significance cut-off of *p* < 0.001; Figure S4: Differentially abundant species-level entities across the CTRL and PKU study groups at the significance cut-off of *p* < 0.001; Figure S5: Differentially abundant species-level entities across the CTRL and PKU study groups and PKU groups with different adherence to diet at the significance cut-off of *p* < 0.001; Figure S6: RDA triplot of the results of the sensitivity analysis of the salivary samples collected from control and type 1 diabetes study group patients. Green hollow triangles represent samples, blue crosses represent the states of a categorical explanatory variables, blue arrows for quantitative explanatory variables with arrowheads indicating their direction of increase, and the taxonomic entities are shown as red plus; Figure S7: RDA triplot of the results of the sensitivity analysis of the salivary samples collected from control and phenylketonuria study group patients. Green hollow triangles represent samples, blue crosses represent the states of a categorical explanatory variables, blue arrows for quantitative explanatory variables with arrowheads indicating their direction of increase, and the taxonomic entities are shown as red plus; Table S1: Selection criteria of patients; Table S2: Pairwise comparisons using Wilcoxon rank sum test and Holm *p*-value adjustment method for alpha diversity metrics; Table S3: Permutation test for homogeneity of multivariate dispersions for beta diversity metrics. Number of permutations used: 999. In the pairwise comparisons, observed *p*-value is shown below diagonal, permuted *p*-value is shown above diagonal.
