# Supplementary material for: Oral Microbiome Traits of Type 1 Diabetes and Phenylketonuria Patients in Latvia
Source: Microorganisms. 2023 May 31;11(6):1471. doi: 10.3390/microorganisms11061471 (PMC10300800; doi:10.3390/microorganisms11061471)
Supplement: Supplementary file 1 [file microorganisms-11-01471-s001.zip › microorganisms-2302362-supplementary.pdf]

**Supplementary Files**

**Table S1**

The questionnaire contained inquiries about:

- General health anamnesis.
- Dental hygiene habits, such as frequency of tooth brushing, flossing, and use of mouthwash and fluoride supplements.
- Frequency of visits to the dentist and dental hygienist.
- Overall satisfaction with their oral health.
- Daily water intake.
- Nutritional habits (frequency of meals, preferred foods at main mealtimes, snacking habits).

PKU patients were additionally asked about:

- Age at which the diagnosis was established.
- Their adherence to PKU diet (intake of Phe-free medical formula and other low-Phe foods, as well as how successfully patients are avoiding high-Phe foods).
- Their current and usual plasma Phe concentrations.

**Figure S1**

**A**

Rarefaction curves before sample rarefaction (A) and after rarefying samples to an even sequencing depth—10,820 sequences per sample (B).

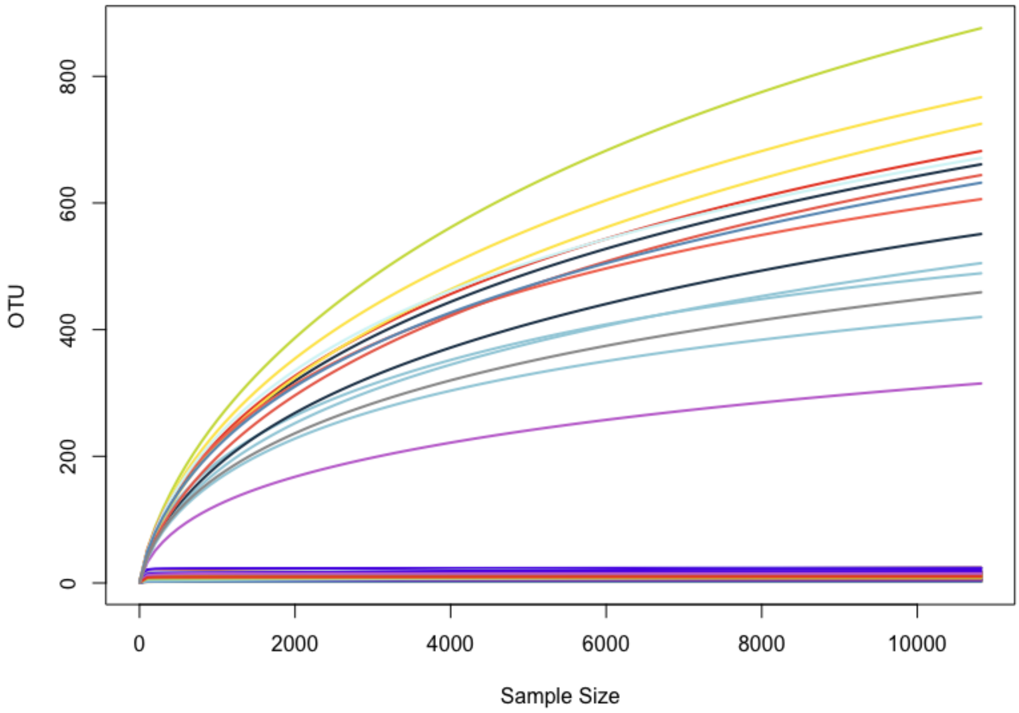


**B**

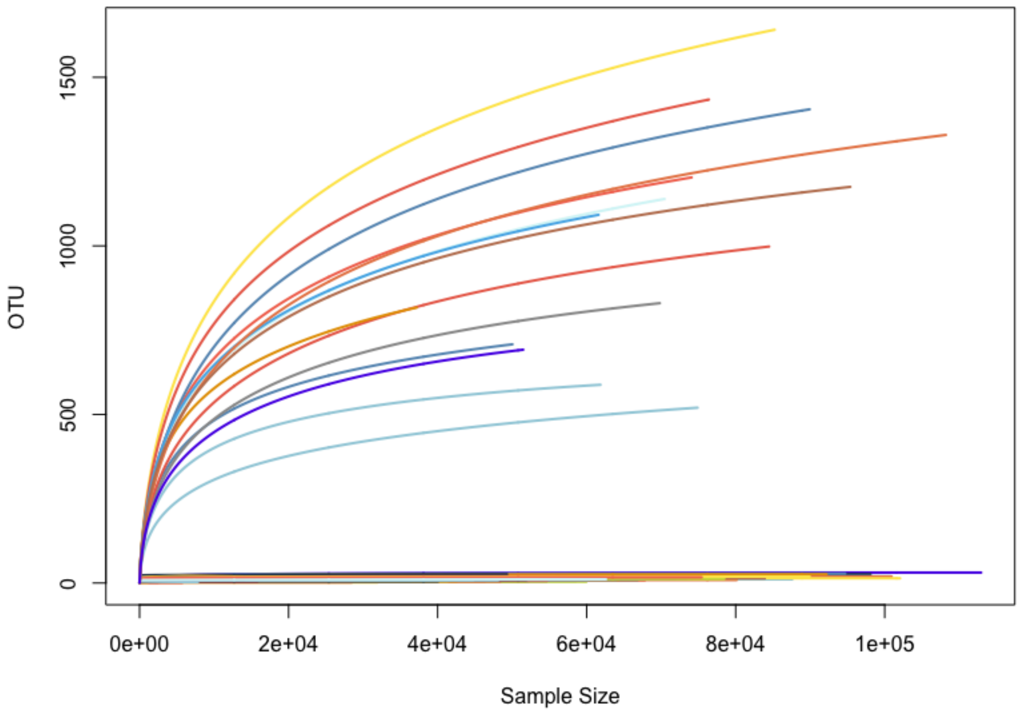


**Table S2**

Pairwise comparisons using Wilcoxon rank sum test and Holm P-value adjustment method for alpha diversity metrics. Abbreviations: CTRL—control group; PKU—phenylketonuria group; T1D—type 1 diabetes group.

**Observed OTUs**

| | CTRL | PKU |
|---|---|---|
| PKU | 0.84 | - |
| T1D | 0.64 | 0.64 |

**Chao1 index**

| | CTRL | PKU |
|---|---|---|
| PKU | 0.79 | - |
| T1D | 0.64 | 0.64 |

**Shannon index**

| | CTRL | PKU |
|---|---|---|
| PKU | 1 | - |
| T1D | 1 | 1 |

**Simpson index**

| | CTRL | PKU |
|---|---|---|
| PKU | 0.94 | - |
| T1D | 0.94 | 0.94 |

**Observed OTUs in PKU group between diets**

| | Strict | Partly |
|---|---|---|
| Strict | 0.52 | - |

| | | |
|---|---|---|
| Partly | 0.58 | 0.79 |

**Chao1 index in PKU group**

| | Strict | Partly |
|---|---|---|
| Strict | 0.50 | - |
| Partly | 0.66 | 0.82 |

**Shannon index in PKU group**

| | Strict | Partly |
|---|---|---|
| Strict | 0.63 | - |
| Partly | 0.63 | 0.88 |

**Simpson index in PKU group**

| | Strict | Partly |
|---|---|---|
| Strict | 0.8 | - |
| Partly | 0.8 | 0.8 |

**<u>Table S3</u>**

Permutation test for homogeneity of multivariate dispersions for beta diversity metrics. Number of permutations used: 999. In the pairwise comparisons, observed P-value is shown below diagonal, permuted P-value is shown above diagonal. Abbreviations: CTRL—control group; PKU—phenylketonuria group; T1D—type 1 diabetes group.

**Weighted UniFrac**

| | CTRL | PKU | T1D |
|---|---|---|---|
| CTRL | - | 0.15400 | 0.807 |
| PKU | 0.15460 | - | 0.141 |
| T1D | 0.77253 | 0.14868 | - |

**Unweighted UniFrac**

| | CTRL | PKU | T1D |
|---|---|---|---|
| CTRL | - | 0.15400 | 0.636 |
| PKU | 0.14971 | - | 0.577 |
| T1D | 0.65039 | 0.59556 | - |

**Figure S2**

Differentially abundant species-level entities across the study groups at the significance cut-off of $p<0.001$. Abbreviations: CTRL—control group; PKU—phenylketonuria group; T1D—type 1 diabetes group.

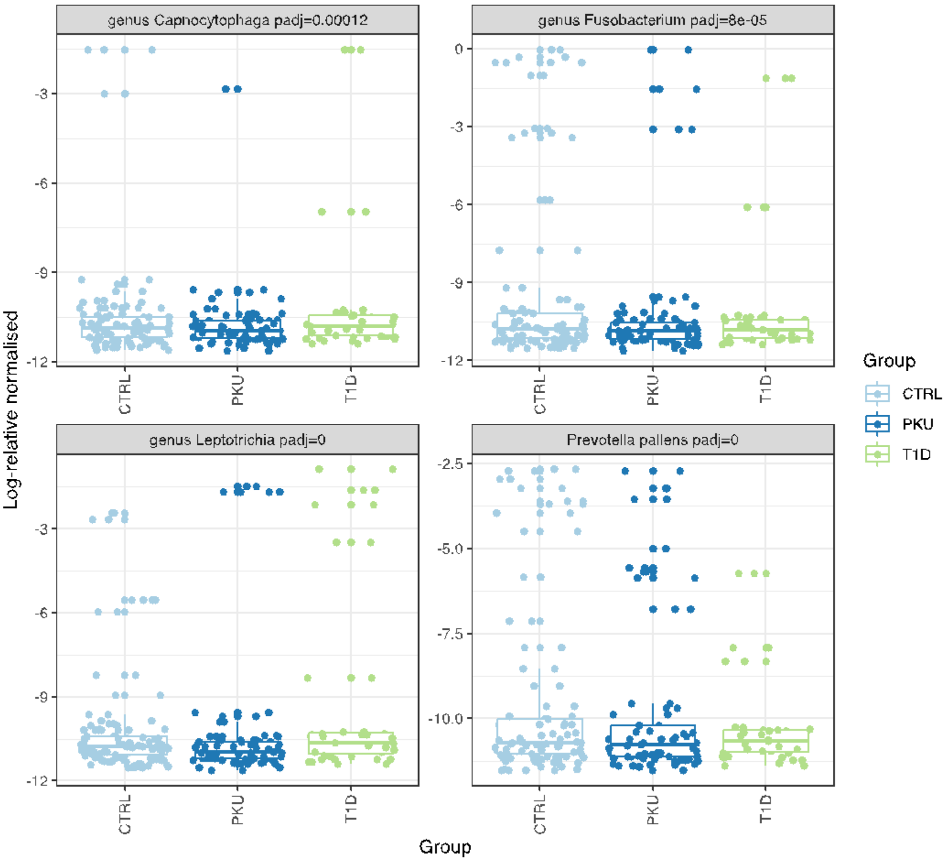

**Figure S3**

Differentially abundant species-level entities across the CTRL and T1D study groups at the significance cut-off of $p<0.001$. Abbreviations: CTRL—control group; T1D—type 1 diabetes group.

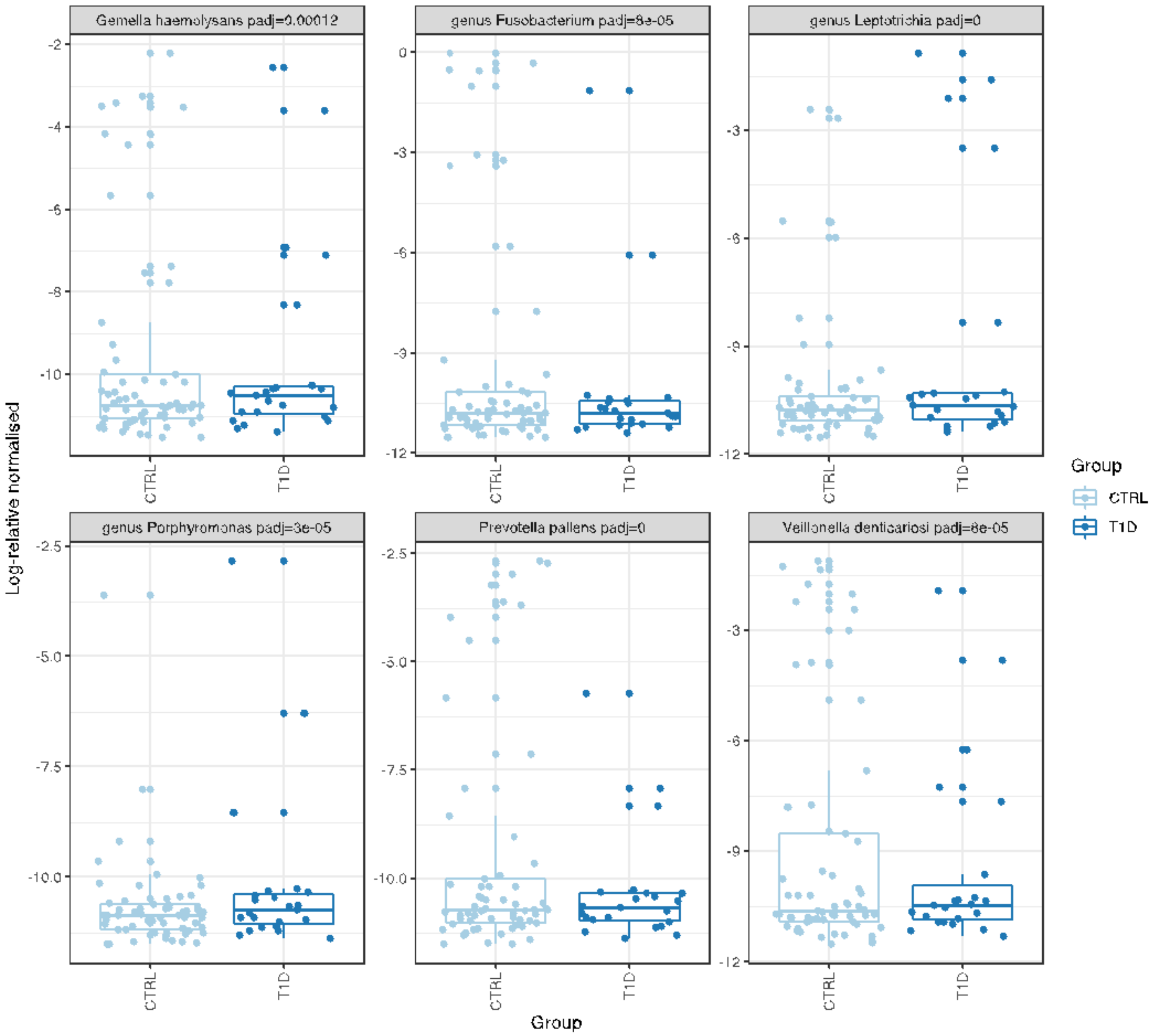

**Figure S4**

Differentially abundant species-level entities across the CTRL and PKU study groups at the significance cut-off of $p<0.001$. Abbreviations: CTRL—control group; PKU—phenylketonuria group.

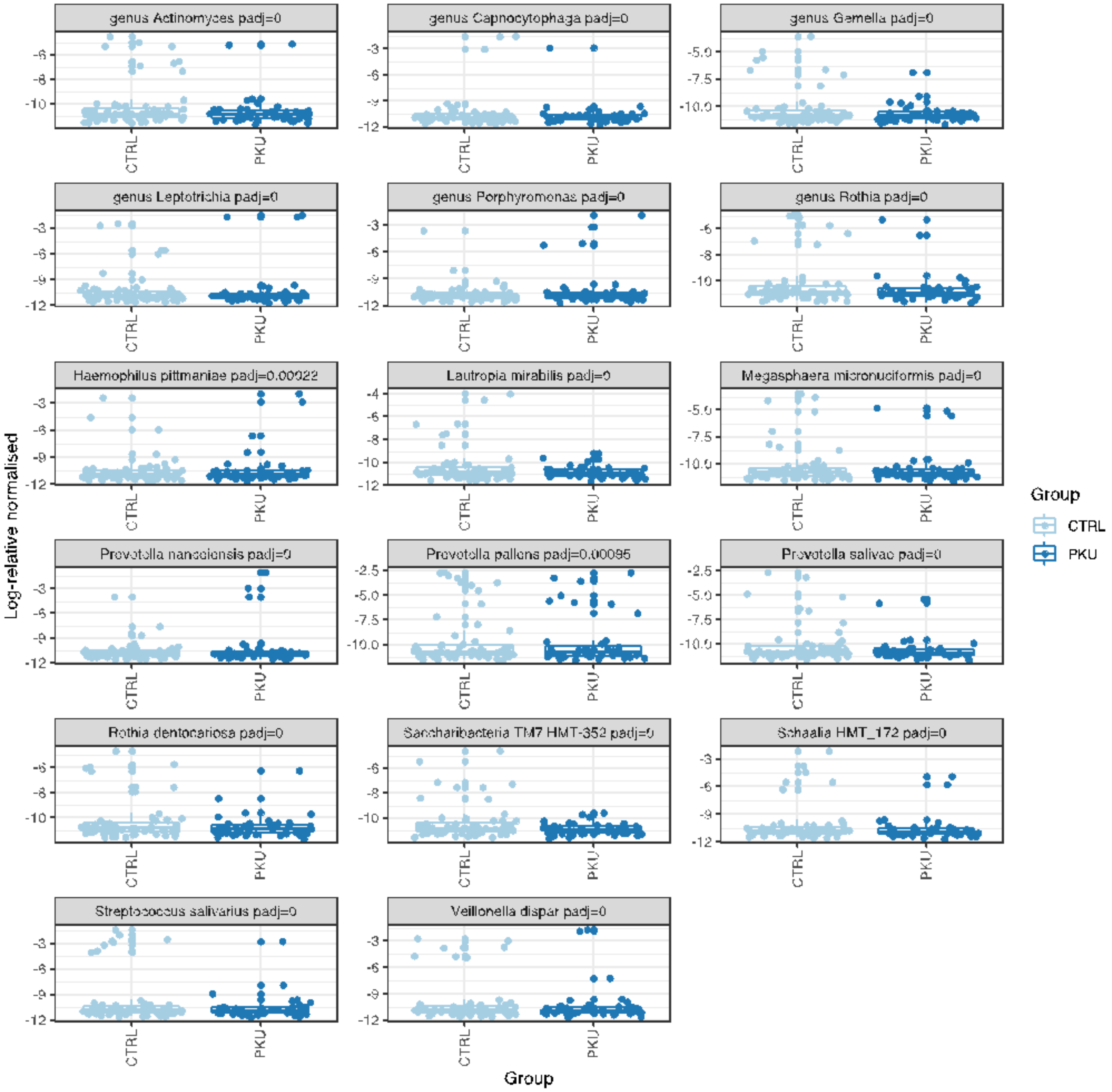

**Figure S5**

Differentially abundant species-level entities across the CTRL and PKU study groups and PKU groups with different adherence to diet at the significance cut-off of $p<0.001$. Abbreviations: CTRL—control group; PKU—phenylketonuria group; PKU-No—patients of phenylketonuria group with no diet regime; PKU-Partly—patients of phenylketonuria group with partial diet regime; PKU-Yes—patients of phenylketonuria group with strict adherence to the diet.

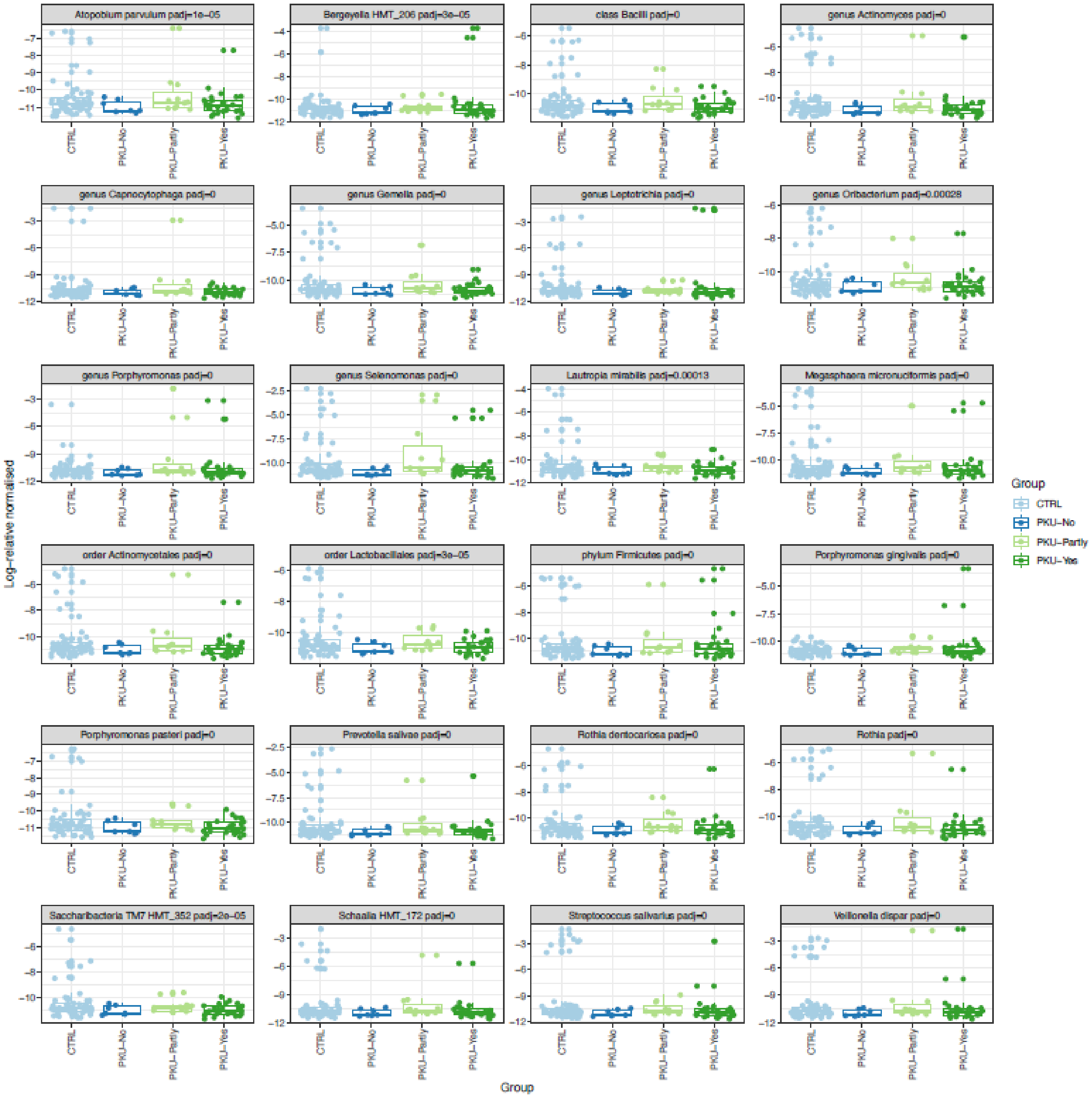

**Figure S6**

RDA triplot of the results of the sensitivity analysis of the salivary samples collected from control and type 1 diabetes study group patients. Green hollow triangles represent samples, blue crosses represent the states of a categorical explanatory variables, blue arrows for quantitative explanatory variables with arrowheads indicating their direction of increase, and the taxonomical entities are shown as red plus.

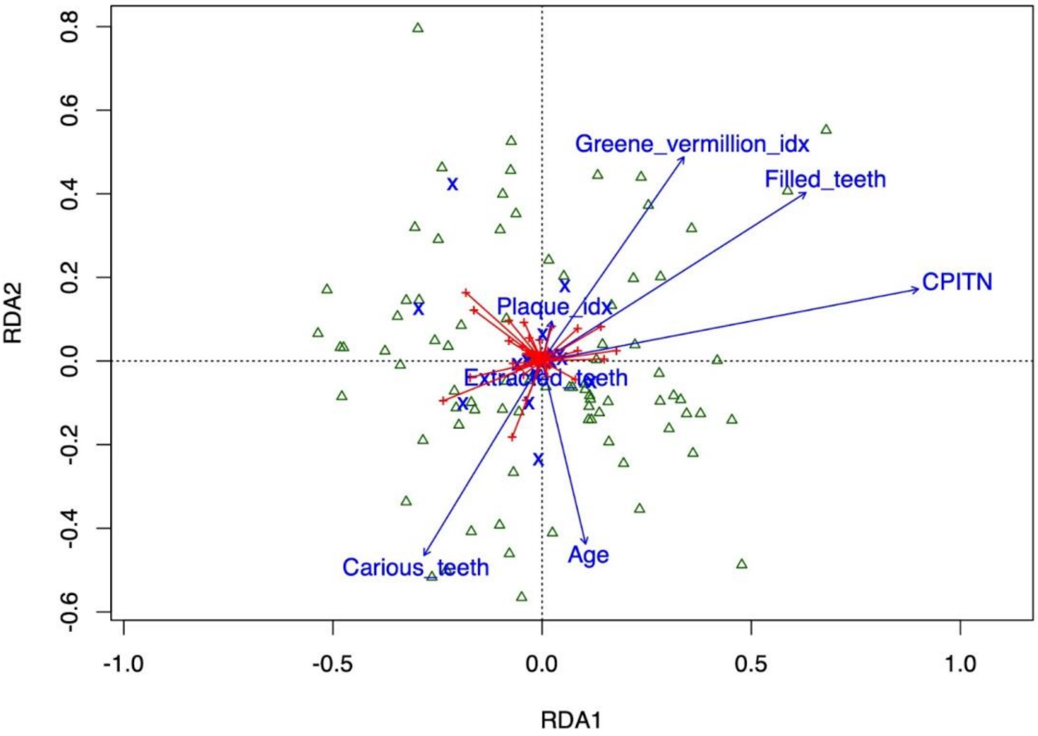


---

**Figure S7**

RDA triplot of the results of the sensitivity analysis of the salivary samples collected from control and phenylketonuria study group patients. Green hollow triangles represent samples, blue crosses represent the states of a categorical explanatory variables, blue arrows for quantitative explanatory variables with arrowheads indicating their direction of increase, and the taxonomical entities are shown as red plus.

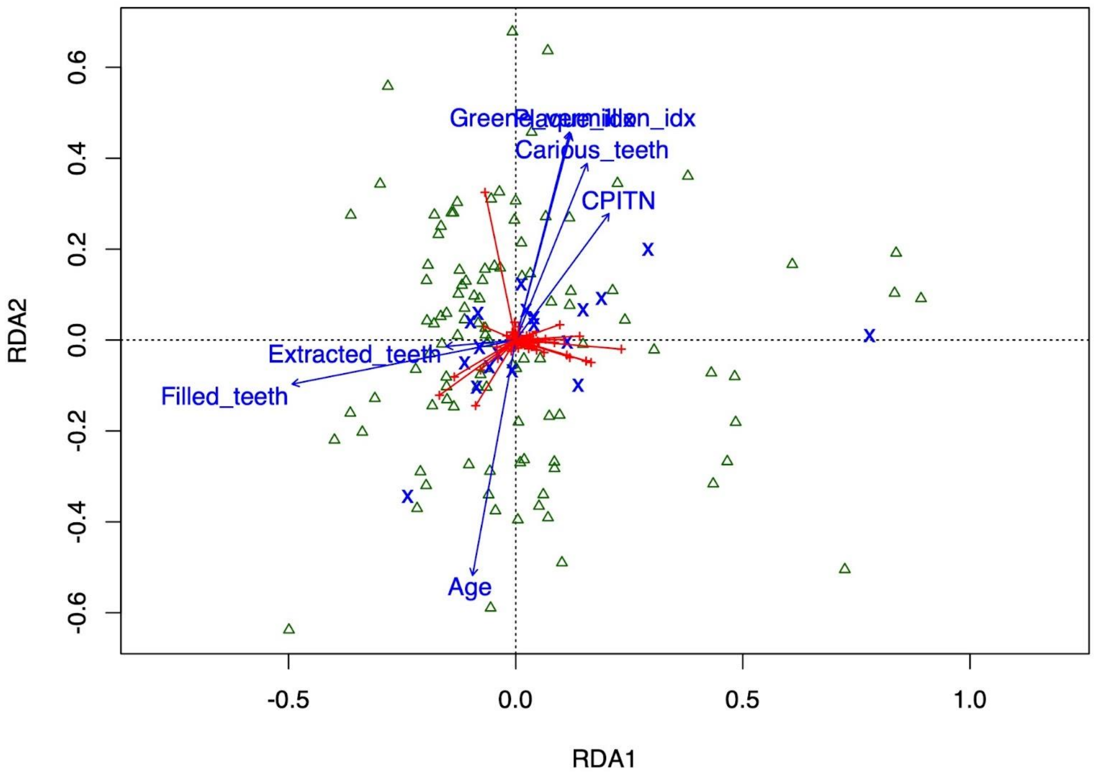
Greene_vermillion_idx
Plaque_idx
Carious_teeth
CPITN
Extracted_teeth
Filled_teeth
Age
RDA1
RDA2
-0.5
0.0
0.5
1.0
-0.6
-0.4
-0.2
0.0
0.2
0.4
0.6
